# Heterogeneity in the Genetic Architecture of Alzheimer disease (AD) across Ancestry Groups

**DOI:** 10.1002/alz70855_107285

**Published:** 2025-12-25

**Authors:** Penelope Benchek, Christiane Reitz, Sven‐Thorsten Dietrich, Nicholas R. Ray, Kara L Hamilton‐Nelson, Gary W Beecham, Jeffery M Vance, Goldie S Byrd, Jiji T. Kurup, Giuseppe Tosto, Adam C. Naj, Rufus O Akinyemi, Jonathan L Haines, Margaret Pericak‐Vance, Richard Mayeux, Li‐San Wang, Lindsay A. Farrer, Gerald D. Schellenberg, William S Bush, Alzheimer's Disease Genetics Consortium (ADGC)

**Affiliations:** ^1^ Department of Population & Quantitative Health Sciences, School of Medicine, Case Western Reserve University, Cleveland, OH, USA; ^2^ Columbia University Irving Medical Center, New York, NY, USA; ^3^ John P. Hussman Institute for Human Genomics, University of Miami Miller School of Medicine, Miami, FL, USA; ^4^ Wake Forest University School of Medicine, Winston‐Salem, NC, USA; ^5^ University of Pennsylvania Perelman School of Medicine, Philadelphia, PA, USA; ^6^ College of Medicine, University of Ibadan, Ibadan, Oyo, Nigeria; ^7^ Department of Biostatistics, Boston University School of Public Health, Boston, MA, USA; ^8^ Department of Population and Quantitative Health Sciences, Case Western Reserve University School of Medicine, Cleveland, OH, USA

## Abstract

**Background:**

In AD genomics research there are known differences in AD prevalence across Non‐Hispanic White (NHW), African American (AA), Asian American (AsA) and Hispanic/Latino (HL) ancestry groups, and paradoxical differences in effect size of *APOE* (larger effects in groups with lower prevalence). These differences highlight abundant heterogeneity in the genetic architecture of AD across ancestries, much of which has not been identified. Estimating the genetic correlation between ancestry groups can explain the extent to which genetic information assessed in one group can inform that of another.

**Method:**

We used the Alzheimer's Disease Genetics Consortium (ADGC) multi‐ancestry TOPMED‐imputed dataset to generate ancestry‐specific heritability estimates for AA (*N* = 6,343), AsA (*N* = 3,197), NHW (*N* = 32,913) and HL (*N* = 1,944) participants and to quantify shared AD genetic architecture. Cross‐ancestry analyses examined high‐quality common variants (minor allele frequency [MAF]>0.05) using a method accounting for ancestry‐specific genetic architecture from Momin, et al. (2023). We first created genetic relationship matrices (GRMs) standardized to ancestry‐specific allele frequencies and scaled to ancestry‐specific allelic effects (i.e., how the variants’ expected heritability contribution depends on MAF). Secondly, we conducted bivariate genomic‐relatedness‐based restricted maximum‐likelihood (GREML) analyses, adjusting for sex, age and principal components to assess the degree of genetic heterogeneity in AD across ancestry pairs.

**Result:**

Overall SNP‐based heritability estimates using this restricted set of variants varied: AA (*h^2^* = 0.13, SE=0.05), NHW (*h^2^* = 0.14, SE=0.01), AsA (*h^2^* = 0.29, SE=0.09) and HL (*h^2^* = 0.48, SE=0.17). The shared genetic component (*r_g_*) of AD was low between AA and HL at 13% (*r_g_* =0.13, SE=0.36), 17% (*r_g_* =0.17, SE=0.35) between AA and AsA, 21% (*r_g_* =0.21, SE=0.30) between HL and AsA and 28% (*r_g_* =0.28, SE=0.15) between NHW and HL. Shared genetics were modestly higher for NHW and AsA at 32% (*r_g_* = 0.32, *se* = 0.14) and for NHW and HL at 42% (*r_g_* = 0.42, SE=0.19).

**Conclusion:**

While AD pathobiology is consistent across affected individuals, the effects of genetic variation may differ greatly across ancestry groups. Our estimates show a low to modest shared genetic component of AD risk across ancestry groups, however these are likely *underestimates* of real effects. These findings highlight that ancestry‐specific genetic contributors for AD remain to be identified.

